# A Pathogenic Presenilin-1 Val96Phe Mutation from a Malaysian Family

**DOI:** 10.3390/brainsci11101328

**Published:** 2021-10-08

**Authors:** Eva Bagyinszky, Gaik-Siew Ch’ng, Mei-Yan Chan, Seong Soo A. An, SangYun Kim

**Affiliations:** 1 Department of Industrial and Environmental Engineering, Graduate School of Environment, Gachon University, Seongnam 13120, Korea; navigator120@gmail.com; 2 Department of Genetics, Penang Hospital, Penang 10990, Malaysia; 3 Department of Genetics, Kuala Lumpur Hospital, Kuala Lumpur 50586, Malaysia; meiyanc@yahoo.com; 4 Department of Bionano Technology, Gachon University, Seongnam 13120, Korea; 5 Department of Neurology, Seoul National University College of Medicine & Neurocognitive Behavior Center, Seoul National University Bundang Hospital, Seongnam 13620, Korea; neuroksy@snu.ac.kr

**Keywords:** Alzheimer’s disease, mutation, whole-exome sequencing, presenilin1

## Abstract

Presenilin-1 (*PSEN1*) is one of the causative genes for early onset Alzheimer’s disease (EOAD). Recently, emerging studies have reported several novel *PSEN1* mutations among Asians. In this study, a *PSEN1* Val96Phe mutation was discovered in two siblings from Malaysia with a strong family history of disease. This is the second report of *PSEN1* Val96Phe mutation among EOAD patients in Asia and in the world. Patients presented symptomatic changes in their behaviors and personality, such as apathy and withdrawal in their 40s. Previous cellular studies with COS1 cell lines revealed the mutation increased the amyloid-β42 (Aβ42) productions. In the present study, whole-exome sequencing was performed on the two siblings with EOAD, and they were analyzed against the virtual panel of 100 genes from various neurodegenerative diseases. In silico modeling was also performed on PSEN1 Val96Phe mutation. This mutation was located on the first transmembrane helix of PSEN1 protein, resulting significant intramolecular stresses in the helices. This helical domain would play a significant role in γ-secretase cleavage for the increased Aβ42 productions. Several other adjacent mutations were reported in this helical domain, including Ile83Thr or Val89Leu. Our study suggested that perturbations in TMI-HLI-TMII regions could also be associated with C-terminal fragment accumulation of APP and enhanced amyloid productions.

## 1. Introduction

Early-onset Alzheimer’s disease (EOAD) occurs before than 65 years of age and represents only a minority of all AD cases (less than 5%). Presenilin1 (*PSEN1*, NC_000014.9) was verified as one of the major causative factors for EOAD. More than 300 pathogenic mutations were found in *PSEN1* (http://www.alzforum.org/mutations/psen-1, accessed on 1 September 2020). Mutations in *PSEN1* may represent clinical heterogeneity, since besides cognitive dysfunctions and memory decline, additional disease phenotypes could present, such as spastic paraparesis, language-and behavioral dysfunctions or Parkinsonism [1]. Patients with *PSEN1* mutation could usually develop disease in their 40s or 50s. In addition, several cases of young onset AD were also reported with clinical phenotypes of cognitive declines in their 30s or even earlier [2,3,4].

Presenilin proteins are members of gamma secretase complex, which plays a role in the cleavage of APP protein. PSEN1 participates as a catalytic subunit of intramembranous aspartyl protease, inducing γ-secretase cleavage at C99 for the production of β-amyloid peptide (Aβ). Aβ peptide could exist in various lengths, such as 43, 42, 40, or 38 amino acids residues. Pathogenic *PSEN1* mutations would modify the γ-secretase activity, resulting in elevated ratio of Aβ42/Aβ40 [3,4]. Alternatively, *PSEN1* mutations could reduce the α-secretase cleavage, resulting in reduced production of Aβ40. In addition, low levels of Aβ40 against Aβ42 may reduce the degree of Aβ42 clearance and enhance the accumulations of Aβ42 [3,4,5]. Elevated Aβ42 and reduced secretion of Aβ species could increase ratio of Aβ42/Aβ40 [5,6,7].

Here, we reported a pathogenic mutation, *PSEN1* Val96Phe (c.286G>T) in a Malaysian family. PSEN1 Val96Phe mutation was initially described in a Japanese family, with similar age of onset [8]. The Malaysian family was briefly mentioned in our previous publication [9]. In the current study, detailed clinical phenotypic presentation and the structure predictions will also be presented. 

## 2. Materials and Methods

Patients provided written informed consent, which allowed the genetic and clinical data to be used for research purposes. A diagnosis of probable AD was carried out, according to the criteria of the National Institute of Neurological and Communicative Disorders and Stroke Alzheimer’ Disease and Related Disorders Association [10]. Detailed clinical phenotypes, family history and imaging data are presented in the Results (Section 3.1).

White blood cells (Buffy coat) have been separated from plasma after centrifugation at 800 g for 30 min. Genomic DNA was extracted using the QIAcube system (Qiagen) and both the quantity and quality of extracted DNA were measured using Nanodrop ND-1000 Spectrometer (NanoDrop, Thermo Fisher Scientific., Seoul, Korea). Whole-exome sequencing (WES) was performed on the two siblings by Novogene Inc (https://en.novogene.com, accessed on 1 September 2020; Hong Kong). A total of 2 μg of genomic DNA used for genetic analysis. After library preparation sequencing was performed on Illumina platform. Whole annotation of data was received by excel file, and sequencing data were sent as a “.bam” file, which were visualized by Integrative Genomics Viewer (IGV) software. Data were analyzed by a “virtual gene panel” of 100 possible genes from different neurodegenerative diseases, including Alzheimer’s disease, Parkinson’s disease, frontotemporal dementia, and prion diseases (Supplementary Table S1 and S2).

Probable pathogenic mutations were discovered by WES and verified by standard sequencing [11], performed by BioNeer Inc. (Dajeon, Korea), using Big Dye Terminator Cyclic sequencing, and data were analyzed using an ABI 3730XL DNA Analyzer (Bioneer Inc., Dajeon, Korea). The data were aligned by NCBI Blast (http://blast.ncbi.nlm.nih.gov/Blast.cgi, accessed on 1 September 2020). Mutations were screened against the reference databases, including Korean Reference Genome Database (KRGDB, http://152.99.75.168/KRGDB/menuPages/intro.jsp, accessed on 1 September 2020), GnomAD (https://gnomad.broadinstitute.org/, accessed on 1 September 2020), and 1000 Genomes (http://www.1000genomes.org/, accessed on 1 September 2020) databases.

In terms of in silico analyses for the potential pathogenic mutations, each variant was analyzed by PolyPhen-2 (http://genetics.bwh.harvard.edu/pph2/, accessed on 1 September 2020), Sorting Intolerant From Tolerant algorithm (SIFT; http://sift.jcvi.org/, accessed on 1 September 2020), PROVEAN (http://provean.jcvi.org/index.php, accessed on 1 September 2020) and ExPASy (https://web.expasy.org/protscale/, accessed on 1 September 2020) tools, which provided the estimation of the putative pathogenic nature, as benign or possibly damaging. Protein structure predictions were carried out by Raptor X (http://raptorx.uchicago.edu/, accessed on 1 September 2020), and the structures of the *PSEN1* Val96Phe variant were compared to the normal X-ray structure [12]. Superimposed images of variant and normal proteins were aligned by Discovery Studio 3.5 Visualizer software (designed by Accerlrys Inc., San Diego, CA, USA).

## 3. Results

### 3.1. Subjects

**Figure 1:**
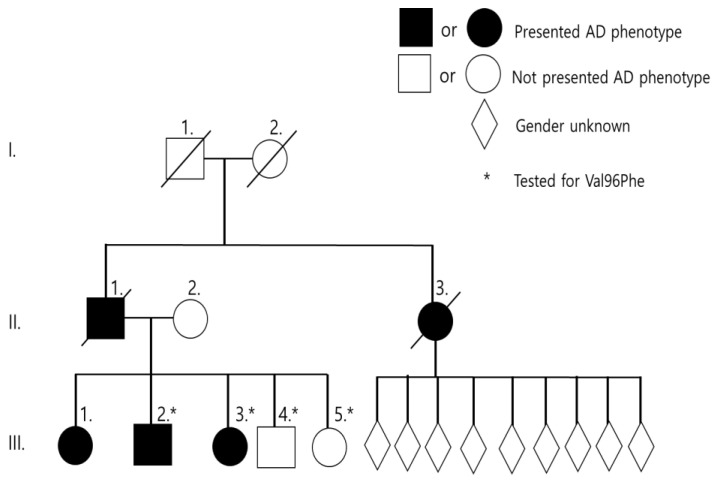
Family tree of Malaysian family.

Two Malaysian siblings (III-2, III-3) were affected with memory loss at the age of 48 and 44 years, respectively. Family history of AD was positive in this family. The eldest sister (III-1) developed AD at 50 years of age but could not be genotyped. Two younger siblings (III-4, III-5) aged 39 and 35 years were asymptomatic at the time of reporting. Clinical phenotype of grandparents (I-1, I-2) remained unclear. The father (II-1) and paternal aunt (II-3) developed dementia at 57 and 50 years, respectively. The paternal aunt (II-3) has nine children, ages ranged from 30–54 years, all of whom were asymptomatic at the time of reporting (Figure 1 and Table 1). Detailed clinical description was provided on III-3 and III-4 patients. Both patients carried the E3/E4 genotype for apolipoprotein E (APOE). 

**Figure 2:**
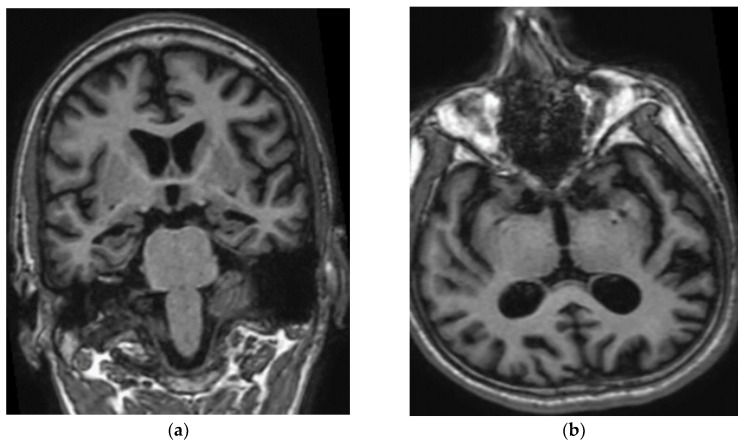
(**a**) Coronal T1W MP RAGE image of brain shows atrophy of bilateral hippocampi, more pronounced on the right side. Widening of the cerebral sulci predominantly in the temporal lobes and both lateral ventricles are also noted. (**b**) Axial T1W MP RAGE image of the brain shows widening of bilateral Sylvian fissure. Dilated occipital horn of both ventricles is also noted.

Patient III-2 experienced medical decline at the age of 47 years. He visited the clinic at the age of 49 years, with no background medical illness. His wife noted that he had short-term memory loss, repeated himself often, and was unable to pay utility bills. The patient also frequently lost items, such as glasses or phone, and had difficulties in driving. He also resigned from his office job related to sales due to stress. At the age of 48 years, speech impairment appeared such as paucity of speech, naming his children, and describing the weather. In addition, he was unable to pronounce words clearly or speak in full sentences. His MMSE on presentation was 16/30. His initial investigations showed normal full blood count, liver and renal function, HbA1c and B12 level, mildly elevated total cholesterol of 5.9 mmol/L, and low folate of 9 nmol/L (12–44 nmol/L). The initial CT brain showed mild cerebral atrophy with no evidence of cerebral infarctions. The MRI brain revealed mild generalized cerebral volume loss prominently involving the temporal and parietal lobes with profound diffuse volume loss over bilateral hippocampal formations and mild volume loss of the parahippocampal gyri. He was prescribed a rivastigmine patch and folate supplements. No leukodystrophy, white matter changes, microbleeds, or cerebral amyloid angiopathy were observed in his brain (Figure 2). A year after the first visit, the patient did not recognize his children and wife, and his speech was no longer meaningful. He started to have repetitive behaviour, motor and sleep disturbances, as well as aggression. He developed apraxia, agnosia, and aphasia. He required help in bathing, dressing, or going to the washroom. His MMSE dropped within a year from 16 to 7. Medications were changed to memantine and donepezil. Two years into follow-up, at the age of 51, he was completely dependent in his activities of daily living. He developed one episode of seizure requiring admission. His MMSE score was 1/30. 

Patient III-3 was first seen at the clinic at age 44 years due to the suspicion of familial early onset Alzheimer’s dementia. She was still working as an office assistant in tax department; however, she was having problems making entries into the computer, and was thus assigned simple tasks such as putting letters into envelopes. She was still able to cook and do house chores but frequently forgot where she put her things. She was no longer able to handle home finances. Her physical examination was unremarkable and her MMSE was 18/30. She had normal full blood count, renal and liver function, calcium, phosphate, HbA1c, thyroid function, cholesterol, and B12 levels. Her folate levels were low at 6.16 nmol/L (12–44 nmol/L). Her MRI brain showed mild reduction in right hippocampal volume, with normal left hippocampus. An asymmetrical hippocampal volume with prominent parietal sulci and ambient cistern is suggestive of early Alzheimer’s disease. She was started on rivastigmine. A year into follow-up, she was still able to work and do house chores but was unable to remember details regarding her children’s education and work. She started developing aggressive behavior and psychotic symptoms. Eating disturbances also appeared in her, and she often refused all food and drink. She also had frequent sleep disturbances. Her MMSE scores declined from 18/30 to 13/30. Rivastigmine was changed to donezepil. Risperidone was started for control of her psychotic symptoms. Two years into follow-up, she was no longer able to work, cook, or do housework and required supervision for showering and dressing. Three years into follow-up, at the age of 47 years, she was fully dependent on her husband and had very limited language. She was still able to move independently, mostly wandering around the house aimlessly. MMSE was reduced to 1/30. Memantine was added to her medications.

### 3.2. Genetic and Structure Findings

**Figure 3:**
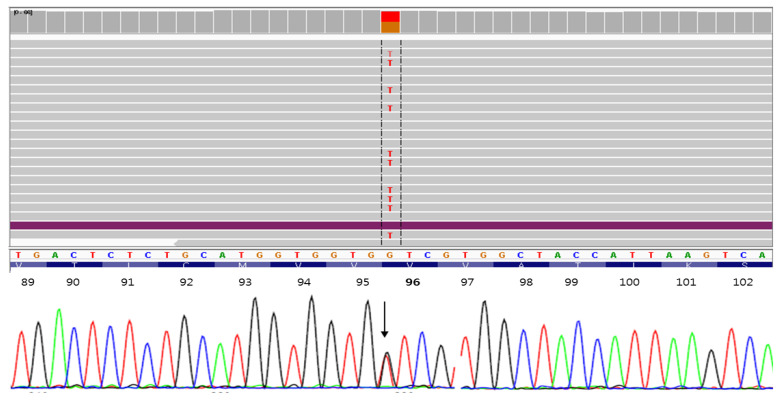
Whole-exome sequencing data of PSEN1 Val96Phe mutation.

**Figure 4:**
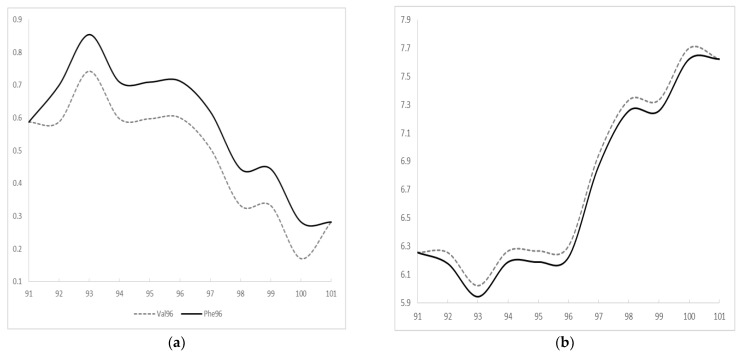
ExPASY prediction on PSEN1 Val96Phe mutation (**a**) hydrophobicity scores and (**b**) polarity scores.

A pathogenic G>T exchange was found in *PSEN1* exon 4 in all two siblings (c.286G>T, III-2, III-3), resulting in valine (Val) to phenylalanine (Phe) exchange at codon 96 (p.Val96Phe, Figure 3). Two asymptomatic siblings were tested too, and III-4 was positive for mutation, but III-5 was negative (Supplementary Figure S1). According to KRGDB database, the mutation was missing among 1100 unaffected Korean individuals. It was neither observed in 1000Genomes nor in GnomAD databases. PolyPhen and SIFT tools predicted the mutation as probably damaging with the score of 1.0 and 0.002, respectively. Multiple sequence alignment by Polyphen2 tool revealed that PSEN1 Val96 may be a conservative residue between different species, since valine was detected in the same residue in the PSEN-like sequence of other animal species. PROVEAN also predicted the mutation as deleterious with the score of −4.643. ExPASY revealed higher hydrophobicity scores for Phe (Val: 0.6; Phe: 0.712, Figure 4a). The polarity score for Phe was slightly reduced in comparison to Val (Val: 6.3; Phe: 6.222, Figure 4b). 

**Figure 5:**
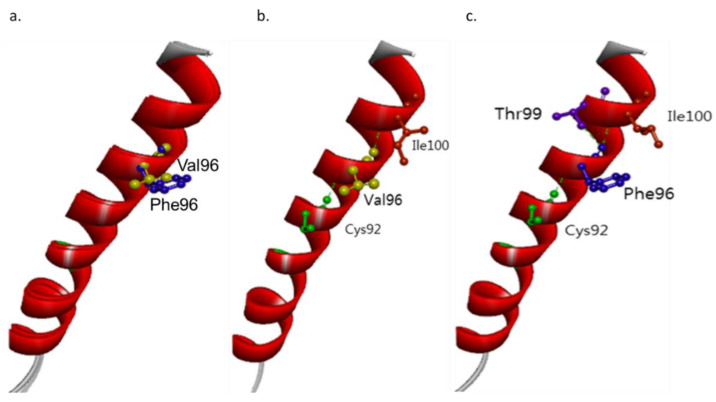
(**a**) 3D model on PSEN1 Val96Phe mutation, compared to the normal PSEN1 protein. (**b**) Intramolecular interactions in PSEN1 Val96. (**c**) Intramolecular interaction for PSEN1 Phe96.

Mutation is located in the first TM helix of PSEN1 protein. Structure predictions revealed the putative disturbances in Helix-I (Figure 5a). Even though both Val and Phe are non-polar amino acids, the slightly higher hydrophobic property of Phe may disturb the helix motion and dynamics of PSEN1. The larger size of benzyl group in Phe may also result in extra stress inside the Helix-I.

Intramolecular interactions may also be changed due to *PSEN1* Val96Phe mutation. Val96 could form a hydrogen bond with Ile100 and Cys92 (Figure 5b). In the case of Phe96, interactions with Cys remained, but an additional hydrogen bond may be seen with Thr99 (Figure 5c). This putative novel hydrogen bond may result in limited helix motion, which could possibly affect the *PSEN1* function. The intra-helical interactions inside TM-I may be stronger due to the mutation, which could potentially result in extra stress in the helix. In addition, it may also disturb the interactions between TM-I and additional TM domains inside PSEN1. 

## 4. Discussion

We reported a known pathogenic *PSEN1* mutation, p.Val96Phe, in a Malaysian family. The family history strongly suggested it to be autosomal dominant, since two siblings (III-2 and III-3) with the mutation were diagnosed with EOAD. In addition, their elder sister (III-1), father (II-1), and aunt (II-3) also developed cognitive-and memory dysfunctions. Their younger brother (III-4) also carried the mutation, but he did not develop any disease phenotype yet. He is most likely in pre-symptomatic stage currently. *PSEN1* Val96Phe was reported previously as a pathogenic mutation in a Japanese family. All affected family members from Japan were diagnosed with EOAD, and the symptoms appeared between 49 and 60 years of age. No detailed clinical description was presented in the previous report from Japan. However, the mutation was segregated with the disease, which was missing in 100 control individuals in Japan [8]. PSEN1 Val96Phe was also missing in different reference databases (GnomAD or 1000Genomes), which may also further prove its absence in unaffected individuals. Both Malaysian and Japanese cases were associated with familial EOAD with a similar age of onset. Mutation was with cloned COS-1 cell line. The elevated Aββ42/totalAβ (1.6 times) levels suggested that the mutation could enhance gamma secretase activity, resulting in elevated Aβ42 levels [13,14].

**Figure 6:**
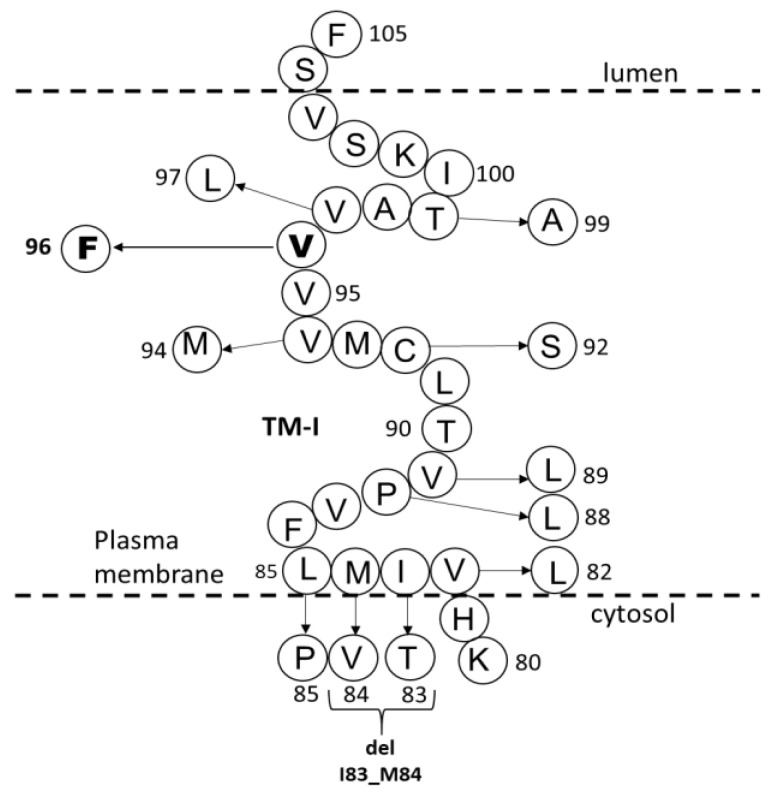
Mutations located in the TM-I of PSEN1. Mutations in TM-I may result in dysfunctions in γ-secretase cleavage by altering the intra-and intermolecular interactions of PSEN1.

PSEN1 Val96Phe is in the first transmembrane domain (TM1) in PSEN1 protein. Several pathogenic mutations were discovered in the TM1 of PSEN1 (Table 2, Figure 6). The age of onset associated with these mutations in TMI could be variable. The majority of patients with the mutations reported the age of onset in their 40s or 50s [15]. In addition, several mutations were associated with younger onset AD, such as *PSEN1* Leu85Pro [16], Pro88Leu [17], Val89Leu [18], and Val97Leu [19]. All patients with these mutations were diagnosed with EOAD. Patients with Val82Leu [20], Val96Phe [8], or Thr99Ala [21] mutations presented typical EOAD symptoms. Other clinical symptoms could also appear, such as behavioral/psychiatric symptoms for patients with Ile83Thr [22], Met84Val [23], or Val89Leu [18] mutations. Spastic paraparesis were prominent for patients with Ile83Thr [22], del_Ile83/Met84 [24,25], or Leu85Pro [16]. Parkinsonism was also discovered in patients with Pro88Leu [17] or Cys92Ser [26]. The following mutations, del_Ile83/Met84 [24,25], Leu85Pro [16], Val96Phe [8,13], Val97Leu [19,27], were transfected into cells for the verification of their pathogenicity. Interestingly, the cloned Val82Leu mutation into CHO cells revealed reduced Aβ42 levels, suggesting its participation in the pathogenic mechanisms [28]. 

TM-I domain was found to be relatively conservative between PSEN1 and PSEN2 for its critical role in γ-secretase activity. Hence, even slight alterations of structure might have larger effects in Aβ metabolism. Experiments of deleting TM-I and TM-II domains or the connecting loop (HL-I) in PSEN1 (Val82-Tyr154) could affect the endoproteolysis of PSEN1, affecting the APP metabolism. The deletion of these domains may result in the accumulation of CTF fragment of APP by delayed γ-secretase processing. A loss of PSEN1 function may also impair the APP trafficking. In addition, the deletion of TM1-HLI-TMII region may also impair the Notch signaling [31,32]. Structure predictions by Queralt et al. (2002) revealed that in normal PSEN1 protein, valine residues in TM-I may be in contact with the hydrophobic residues in TM-VII. Mutations in TM-I may increase the distance between TM-I and TM-VII, resulting in additional stress inside the PSEN1 protein [18]. All of these findings suggested that mutations in the TM-I may be responsible pathogenic mutations in EOAD. Cell studies from different mutations (including Val96Phe) suggested that TM-I may play a significant role in APP trafficking and amyloid peptide cleavage.

## 5. Conclusions

In this study, a pathogenic *PSEN1* mutation, Val96Phe, was described in a Malaysian family. The mutation was initially discovered in a Japanese family in 1996. The Malaysian case revealed a strong family history of disease, since at least two family members with EOAD carried the mutation, and other relatives were affected with EOAD. This report may add additional clinical details, associated with AD patients, binding PSEN1 Val96Phe mutation. This is a second pathogenic PSEN1 mutation, which was described in a Malaysian family [33]. Due to the algorithm of Guerreiro et al. (2010) PSEN1 Val96Phe is a pathogenic mutation. The mutation was missing in different reference databases (1000Genomes, GnomAD), suggesting that it may not be present in individuals without any neurodegenerative disease. Multiple sequence alignment revealed that Val96 is a conservative residue among vertebrates. Additionally, PSEN1 Val96Phe is located in a region which was verified as conservative between PSEN1 and PSEN2 [34]. In addition, PSEN1 Val96Phe appeared in patients from two independent families affected with EOAD [34]. Our in silico prediction suggested that mutation could possibly disturb the intramolecular interactions inside the PSEN1. In addition, it could probably result in abnormalities in PSEN1 and γ-secretase functions by resulting in stress between PSEN1 TM-I and TM-VIII. Mutations in TM-I could also potentially affect the endoproteolysis of PSEN1 protein, resulting in disturbances in APP cleavage, and elevated Aβ42 productions [31,32]. The limitations of this study were that we were unable to perform cerebrospinal fluid (CSF) amyloid biomarker study since patients refused CSF analysis. Additionally, full segregation analysis could not be performed since several affected family members passed away and/or no sample was available from them. Additionally, the currently asymptomatic cousins of the patient refused the genetic test. Furthermore, no in vitro study was performed on this mutation by our group. However, in the future, we will perform in vitro analyses on the mutation in additional cell lines, such as HEK-293.

## Figures and Tables

**Table 1:** Clinical details of Malaysian family with EOAD.

	Age of Onset	Memory Loss	Cognitive Deficits	Deficits in Spatial Awareness	Behavioral and Personality Change	Seizures	V96F Mutation
II-1	57	+	+	+	+	-	NA
II-3	50	+	+	+	+	-	NA
III-1	50	+	+	-	-	-	NA
III-2	48	+	+	+	+	+	Positive
III-3	44	+	+	-	+	-	Positive
III-4	No symptoms	-	-	-	-	-	Positive
III-5	No symptoms	-	-	-	-	-	Negative

**Table 2:** Mutations, described in TM-I domain of PSEN1. Majority of mutations were associated with relatively young onset and positive family history of disease.

Mutation	Clinical Symptoms	Age of Onset (Year)	Family History	Functional Studies	References
Val82Leu	EOAD	53–58	Positive (2 French family)	HEK293: 1.5 times elevated Aβ42/Aβ40 CHO: 1.4 times reduced CHO-APP695	[20]
Ile83Thr	EOAD, behavioral symptoms, depression, hallucinations	55–64	Probable positive (Tunisian)	NA	[22]
del_Ile83/Met84	EOAD, spastic paraparesis, cotton wool plaques, cerebral amyloid antipathy	34–38	Positive (Scottish)	HEK293: 4.8 times elevated Aβ42/Aβ40 H4: 2.6 times higher Aβ42/Aβ40	[24,25]
Met84Val	EOAD, psychotic symptoms	49–57	Positive (Italian)	NA	[23,29]
Leu85Pro	EOAD, spastic paraparesis	26	De novo (Japan)	HEK293: 1.9 times elevated Aβ42/Aβ40	[16]
Pro88Leu	EOAD, myoclonus, Parkinsonism, apraxia	20s	Unknown (China)	Increased the long amyloid peptides	[17]
Val89Leu G>C	EOAD	Late 30s	Unknown (China)	NA	[24]
Val89>Leu G>T	EOAD with personality changes	46–51	Familial (Spain)	NA	[18]
Cys92Ser	EOAD, parkinsonism, hallucination	49–70	Familial (Italy)	Fibroblast cells: elevated Aβ42 levels	[26]
Val94Met	EOAD	53	De novo (Columbia)	NA	[30]
Val96Phe	EOAD	44–57	Familial (Japan, Malaysia)	elevated Aβ42/totalAβ in COS1 cells	[8,13], our case
Val97Leu	EOAD	Late 30s, early 40s	Familial (China)	SH-SY5Y cells: elevated intracellular and extracellular Aβ42	[19,27]
Thr99Ala	EOAD	43	De novo (Japan)	NA	[21]

## Data Availability

Data available on request due to restrictions eg privacy or ethical.

## Supplementary Materials

The following are available online at https://www.mdpi.com/article/10.3390/brainsci11101328/s1, Figure S1: Sequencing data of family members, Table S1: Genes, involved in the gene panel, Table S2: (a). Variants, found in III-3 (b). variants found in III-4.
