# Supplementary material for: A Pathogenic Presenilin-1 Val96Phe Mutation from a Malaysian Family
Source: Brain Sci. 2021 Oct 8;11(10):1328. doi: 10.3390/brainsci11101328 (PMC8534005; doi:10.3390/brainsci11101328)
Supplement: Supplementary file 1 [file brainsci-11-01328-s001.zip › brainsci-1394263-supplementary.pdf]

Supplementary Materials

Table S1. Genes, involved in the gene panel.

Dx	No	Genes, involved in the gene panel
AD	40	ABCA7, ACE, ADAM10, APP, BACE1, BIN1, CASS4, CD2AP, CD33, CDH12, CDH18, CR1, CTNNA3, CLU, DNMBP, EPHA1, DSG2, HLA-DRB5, HLA-DRB1, INPP5D, LPR6, MEF2C, MTHFD1L, MS4A4A, MS4A6E, NME8, PICALM, PSEN1, PSEN2, PLD3, PTK2B, RIN3, S100A9, SLC24A4, SORL1, TM2D3, TOMM40, ZCWPW1
PD	22	ACMSD, BST1, FBXO7, FGF20, GAK, GBA, GIGYF2, GPNMB, HIP1R, LAMP3, LRRK2, PARK2, PARK7, PARK9, PINK1,PLA2G6, SNCA, STBD1, STK39, STX1B, SYT11, VPS35
ALS &FTD	30	ANG, ALS2, ATXN1, ATXN2, CHMP2B, CCNF, DAO, DCTN1, EWSR1, FIG4, FUS, GRN, HNRNPA1, HNRNPA2B1, MAPT, NEK1, OPTN, PFN1, PPT1, SETX, SIGMAR1, SOD1, SQSTM1, TAF15, TARDBP, TMEM106B, TBK1, UBQLN2, VAPB, VCP
Other disease	8	CTSA, CYP7B1, CSF1R, HTRA1, NOTCH3, SPAST, SPG11, PRNP

Table S2. (a). Variants, found in III-3 (**b**). variants found in III-4.

a.

Gene	Mutation	RsID	1000g_eas	1000g_all	ExAC_ALL	ExAC_EAS	SIFT	Polyphen2
ABCA7	E188G	rs3764645	0.4355	0.399561	0.4838	0.4379	0.647,T	0.358,B
	R1349Q	rs3745842	0.3482	0.390575	0.4433	0.3601	0.546,T	0.008,B
	G1527A	rs3752246	0.6478	0.825479	0.8405	0.6552	0.877,T	0.0,B
	A2045S	rs4147934	0.4325	0.605032	0.7317	0.544	0.962,T	0.057,B
ALS2	V368M	rs3219156	1	0.896565	0.9106	0.9999	0.191,T	0.006,B
ATP13A2	A1072T	rs3170740	0.246	0.33127	0.5065	0.3343	1.0,T	0.001,B
ATXN1	Q215H	rs184327938	NA	NA	0.0004	0.005	0.316,T	0.0,B
	Q213H	rs3817753	NA	NA	0.0031	0.0081	0.072,T	0.0,B
	H211Q	rs59310777	0.3879	0.266374	0.0636	0.1175	0.297,T	0.0,B
ATXN2	S248N	rs7969300	0.5228	0.179513	0.0905	0.5838	0.136,T	0.0,B
	p.188_189del	rs10560189	1	0.951078	0.5912	0.53	NA	NA
BACE1	C412R	rs539765	1	1	0.9997	1	1.0,T	0.0,B
CR1	H1658R	rs2274567	0.3274	0.294329	0.251	0.2821	0.897,T	0.995,D
	T1858M	rs3737002	0.3264	0.248802	0.275	0.3408	0.021,D	1.0,D
	T2060S	rs4844609	1	0.995008	0.9853	1	1.0,T	0.003,B
	I2065V	rs6691117	0.3433	0.493411	0.3341	0.3014	1.0,T	0.001,B
	P2277R	rs3811381	0.3224	0.26278	0.2403	0.278	0.446,T	0.643,P
	T2419A	rs2296160	0.6885	0.828075	0.8159	0.6571	1.0,T	0.0,B
CSF1R	H362R	rs10079250	0.381	0.153355	0.1192	0.3644	0.061,T	0.255,B
CTNNA3	S596N	rs4548513	0.4187	0.485024	0.412	0.4324	1.0,T	0.0,B
CTSA	p.29_29del	rs10582052	0.5784	0.649361	0.6432	0.6281	NA	NA
DSG2	R773K	rs2278792	0.4643	0.240016	0.2676	0.4748	0.383,T	0.026,B
EPHA1	M900V	rs6967117	1	0.960264	0.9366	0.9988	1.0,T	0.0,B
	Q494H	rs375144213	NA	NA	5.77E-05	0	0.497,T	0.996,D
	V160A	rs4725617	0.9921	0.94349	0.9284	0.9914	0.246,T	0.0,B
FBXO7	M115I	rs11107	0.6915	0.48742	0.44	0.6908	1.0,T	0.0,B
FGF20	G116R	rs3793405	0.0179	0.0039936	0.0014	0.0156	0.001,D	0.994,D
FIG4	V654A	rs9885672	0.4077	0.447883	0.2753	0.4072	0.833,T	0.0,B
GIGYF2	P460T	rs2289912	0.2302	0.0830671	0.0524	0.2291	0.375,T	0.756,P

	p.P1210fs	rs371622656	NA	NA	0.0688	0.2392	NA	NA
	p.1210_1211 del	rs10555297	0.6022	0.559105	0.5753	0.5611	NA	NA
	p.P1210fs	rs775324034	NA	NA	0.174	0.3959	NA	NA
LAMP3	I318V	rs482912	0.4712	0.496406	0.6483	0.5081	1.0,T	0.0,B
LRP6	V1062I	rs2302685	0.9196	0.885583	0.8474	0.9296	1.0,T	0.0,B
LRRK2	R50H	rs2256408	1	0.969249	0.9911	1	1.0,T	0.0,B
	S1647T	rs11564148	0.3373	0.285942	0.2983	0.3341	0.953,T	0.0,B
MAPT	Y441H	rs2258689	0.628	0.312899	0.2752	0.6411	0.978,T	0.001,B
MS4A4A	K33E	rs10750931	0.1349	0.154553	0.1435	0.1143	0.084,T	0.583,P
	M159V	rs6591561	0.3849	0.329073	0.2965	0.3701	0.837,T	0.0,B
NME8	C208R	rs10250905	0.5466	0.743411	0.7383	0.5651	0.046,D	0.001,B
NOTCH3	A2223V	rs1044009	0.5724	0.629393	0.7591	0.6421	0.175,T	0.001,B
OPTN	M98K	rs11258194	0.1359	0.0786741	0.0452	0.1193	0.925,T	0.001,B
	K322E	rs523747	1	0.993411	0.9973	1	1.0,T	0.0,B
PINK1	N521T	rs1043424	0.3571	0.300519	0.2974	0.3596	0.247,T	0.022,B
PSEN1	V96F	rs63750601	NA	NA	NA	NA	0.002,D	1.0,D
RIN3	H215R	rs3829947	0.2748	0.442492	0.4877	0.2729	0.222,T	0.0,B
	T425M	rs3742717	0.4415	0.292532	0.2385	0.4625	0.08,T	0.293,B
	p.967_967del	rs570458246	NA	NA	0.6674	0.7923	NA	NA
SETX	S2612G	rs3739927	0.3591	0.163538	0.0852	0.3901	0.751,T	0.0,B
	I2587V	rs1056899	0.6835	0.538738	0.3926	0.733	1.0,T	0.0,B
	T1855A	rs2296871	0.6379	0.443091	0.2758	0.7029	0.984,T	0.0,B
	I1386V	rs543573	0.3621	0.55611	0.7252	0.2984	0.872,T	0.0,B
	G1252R	rs1183768	0.3621	0.55611	0.7251	0.2982	0.133,T	0.796,P
	D1192E	rs1185193	0.3681	0.640575	0.7635	0.3062	0.377,T	0.004,B
	A660G	rs882709	0.4117	0.213658	0.1211	0.4524	0.008,D	0.728,P
SIGMAR1	R208W	rs11559048	0.0268	0.0129792	0.0078	0.0289	0.014,D	1.0,D
SORL1	Q1074E	rs1699107	1	0.984824	0.9949	1	1.0,T	0.0,B
	V1967I	rs1792120	1	0.979433	0.9953	1	1.0,T	0.0,B
SPG11	M2132T	rs771913148	NA	NA	5.054E-05	0.0007	0.002,D	1.0,D
SYT11	Q48H	rs822522	1	0.954673	0.988	1	0.866,T	0.0,B
TM2D3	L6R	rs2939587	1	0.993411	0.9798	0.9999	0.466,T	0.0,B

b.

Gene	Mutation	RsID	1000g_eas	1000g_all	ExAC_ALL	ExAC_EAS	SIFT	Polyphen2
ALS2	V368M	rs3219156	1	0.896565	0.9106	0.9999	0.191,T	0.006,B
ATP13A2	A1072T	rs3170740	0.246	0.33127	0.5065	0.3343	1.0,T	0.001,B
ATXN1	Q213H	rs3817753	NA	NA	0.0031	0.0081	0.072,T	0.0,B
	H211Q	rs59310777	0.3879	0.266374	0.0636	0.1175	0.297,T	0.0,B
	p.224_226del	rs754954093	NA	NA	0.0042	0.0275	NA	NA
ATXN2	S248N	rs7969300	0.5228	0.179513	0.0905	0.5838	0.136,T	0.0,B
	S220F	rs562996744	0.001	0.0002	NA	NA	0.007,D	0.842,P
	p.188_189del	rs10560189	1	0.951078	0.5912	0.53	NA	NA
BACE1	C412R	rs539765	1	1	0.9997	1	1.0,T	0.0,B
BST1	R145Q	rs2302464	0.2232	0.079074	0.0485	0.2057	0.118,T	1.0,D
CASS4	P660S	rs35031530	0.2381	0.146765	0.0536	0.2591	0.286,T	0.001,B
CD33	A14V	rs12459419	0.1855	0.210663	0.2939	0.1792	0.083,T	0.766,P
CDH12	V68M	rs4371716	0.1438	0.36222	0.2988	0.1522	0.468,T	0.0,B
CR1	H1658R	rs2274567	0.3274	0.294329	0.251	0.2821	0.897,T	0.995,D
	T1858M	rs3737002	0.3264	0.248802	0.275	0.3408	0.021,D	1.0,D

	T2060S	rs4844609	1	0.995008	0.9853	1	1.0,T	0.003,B
	I2065V	rs6691117	0.3433	0.493411	0.3341	0.3014	1.0,T	0.001,B
	P2277R	rs3811381	0.3224	0.26278	0.2403	0.278	0.446,T	0.643,P
	T2419A	rs2296160	0.6885	0.828075	0.8159	0.6571	1.0,T	0.0,B
CSF1R	H362R	rs10079250	0.381	0.153355	0.1192	0.3644	0.061,T	0.255,B
CTNNA3	S596N	rs4548513	0.4187	0.485024	0.412	0.4324	1.0,T	0.0,B
CTSA	p.29_29del	rs10582052	0.5784	0.649361	0.6432	0.6281	NA	NA
DSG2	R773K	rs2278792	0.4643	0.240016	0.2676	0.4748	0.383,T	0.026,B
EPHA1	M900V	rs6967117	1	0.960264	0.9366	0.9988	1.0,T	0.0,B
	Q494H	rs375144213	NA	NA	5.77E-05	NA	0.497,T	0.996,D
	V160A	rs4725617	0.9921	0.94349	0.9284	0.9914	0.246,T	0.0,B
FBXO7	G6E	rs9621461	0.0615	0.065096	0.0888	0.0524	0.7,T	0.001,B
	M115I	rs11107	0.6915	0.48742	0.44	0.6908	1.0,T	0.0,B
FGF20	G116R	rs3793405	0.0179	0.003994	0.0014	0.0156	0.001,D	0.994,D
FIG4	V654A	rs9885672	0.4077	0.447883	0.2753	0.4072	0.833,T	0.0,B
GIGYF2	P460T	rs2289912	0.2302	0.083067	0.0524	0.2291	0.375,T	0.756,P
	p.1210_1211 del	rs10555297	0.6022	0.559105	0.5753	0.5611	NA	NA
GPNMB	S294F	rs35499907	0.13	0.039337	0.0123	0.1208	0.003,D	1.0,D
HIP1R	V782M	rs2271051	0.2262	0.158546	0.0988	0.237	0.53,T	0.0,B
LAMP3	I318V	rs482912	0.4712	0.496406	0.6483	0.5081	1.0,T	0.0,B
LRP6	V1062I	rs2302685	0.9196	0.885583	0.8474	0.9296	1.0,T	0.0,B
LRRK2	R50H	rs2256408	1	0.969249	0.9911	1	1.0,T	0.0,B
	S1647T	rs11564148	0.3373	0.285942	0.2983	0.3341	0.953,T	0.0,B
	M2397T	rs3761863	0.4603	0.551717	0.624	0.4731	0.466,T	0.0,B
MAPT	Y441H	rs2258689	0.628	0.312899	0.2752	0.6411	0.978,T	0.001,B
MS4A4A	M159V	rs6591561	0.3849	0.329073	0.2965	0.3701	0.837,T	0.0,B
NME8	R43K	rs2722372	0.1815	0.245208	0.2301	0.1922	1.0,T	0.0,B
	C208R	rs10250905	0.5466	0.743411	0.7383	0.5651	0.046,D	0.001,B
NOTCH3	A2223V	rs1044009	0.5724	0.629393	0.7591	0.6421	0.175,T	0.001,B
OPTN	M98K	rs11258194	0.1359	0.078674	0.0452	0.1193	0.925,T	0.001,B
	K322E	rs523747	1	0.993411	0.9973	1	1.0,T	0.0,B
PINK1	N521T	rs1043424	0.3571	0.300519	0.2974	0.3596	0.247,T	0.022,B
PSEN1	V96F	rs63750601	NA	NA	NA	NA	0.002,D	1.0,D
RIN3	T425M	rs3742717	0.4415	0.292532	0.2385	0.4625	0.08,T	0.293,B
	R599H	rs200221185	0.001	0.0002	0.0002	0.0023	0.002,D	1.0,D
SETX	S2612G	rs3739927	0.3591	0.163538	0.0852	0.3901	0.751,T	0.0,B
	I2587V	rs1056899	0.6835	0.538738	0.3926	0.733	1.0,T	0.0,B
	T1855A	rs2296871	0.6379	0.443091	0.2758	0.7029	0.984,T	0.0,B
	I1386V	rs543573	0.3621	0.55611	0.7252	0.2984	0.872,T	0.0,B
	G1252R	rs1183768	0.3621	0.55611	0.7251	0.2982	0.133,T	0.796,P
	D1192E	rs1185193	0.3681	0.640575	0.7635	0.3062	0.377,T	0.004,B
	A660G	rs882709	0.4117	0.213658	0.1211	0.4524	0.008,D	0.728,P
SORL1	Q1074E	rs1699107	1	0.984824	0.9949	1	1.0,T	0.0,B
	V1967I	rs1792120	1	0.979433	0.9953	1	1.0,T	0.0,B
SPG11	K1534R	rs377348534	NA	NA	1.65E-05	0	0.965,T	0.002,B
	F463S	rs3759871	0.4653	0.47484	0.4659	0.4674	0.343,T	0.066,B
STBD1	p.T276fs	rs762797442	NA	NA	0.0001	0.0007	NA	NA
SYT11	Q48H	rs822522	1	0.954673	0.988	1	0.866,T	0.0,B
TM2D3	L6R	rs2939587	1	0.993411	0.9798	0.9999	0.466,T	0.0,B
TMEM106B	T185S	rs3173615	0.6508	0.595048	0.4916	0.6538	0.214,T	0.043,B

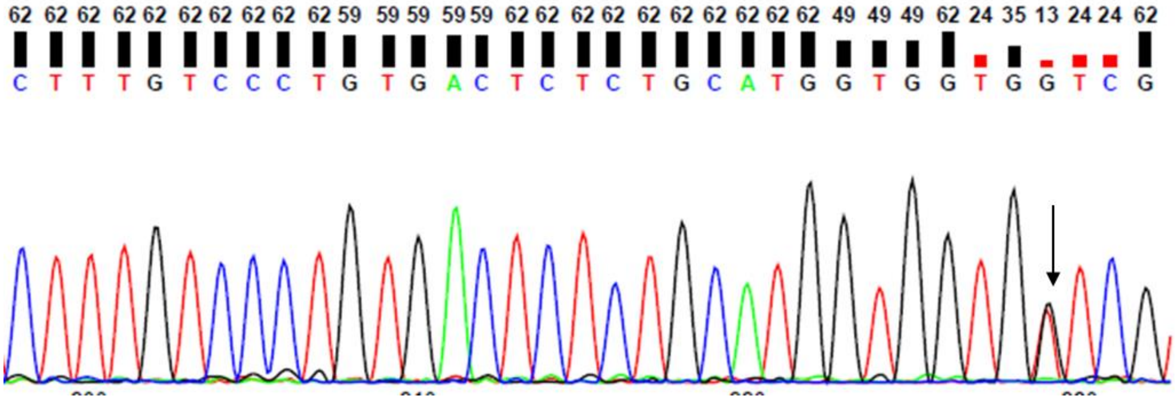

DNA240/18

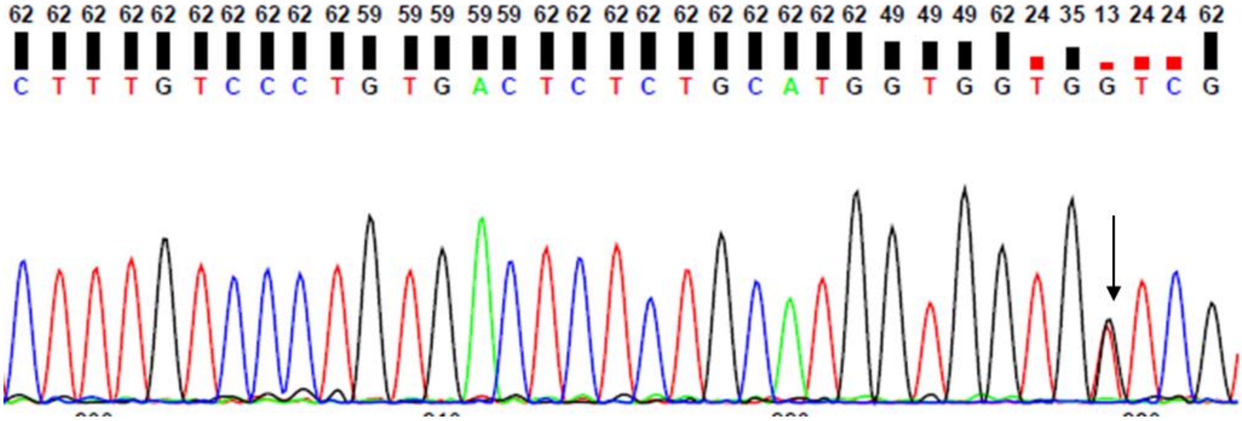

427/19: III-5

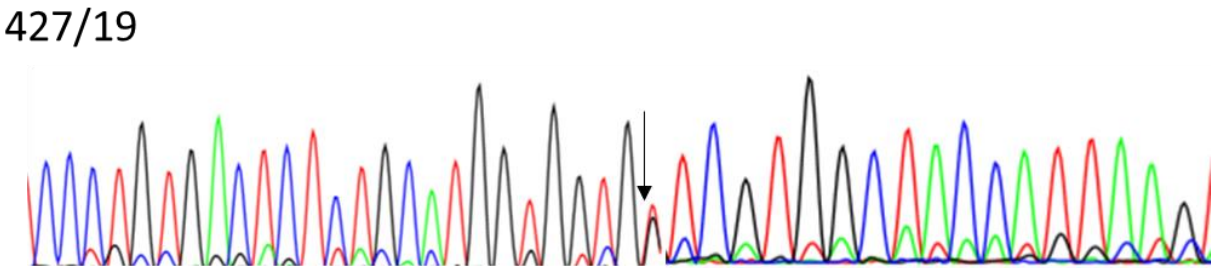

426/16 III-6

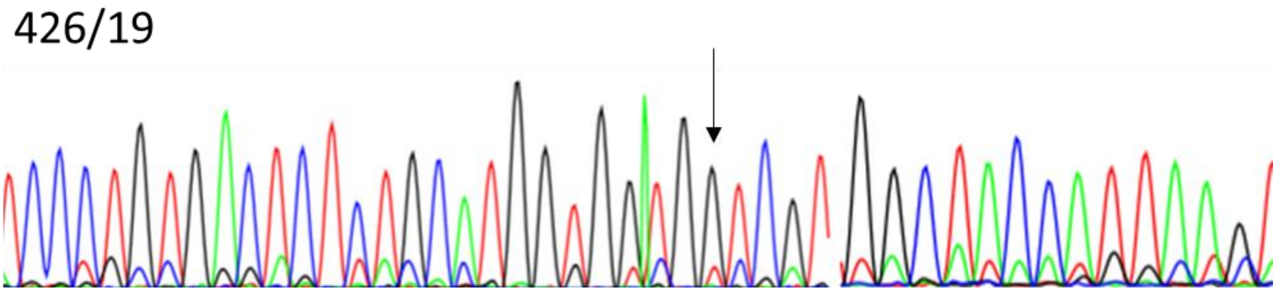

Figure S1. Sequencing data of family members.
